# Acceptability of reducing sedentariness using a mobile-phone application based on ‘if then’ plans for people with psychosis: A focus-group study conducted in North West England, UK

**DOI:** 10.1177/00207640221102733

**Published:** 2022-06-07

**Authors:** Rachel Bailey, Y Kiera Bartlett, Lamiece Hassan, Christopher J Armitage, Charlotte Stockton-Powdrell, Matthew Machin, Shon Lewis, Tracy Epton

**Affiliations:** 1 Division of Psychology & Mental Health, Manchester Centre for Health Psychology, University of Manchester, Manchester, Greater Manchester, UK; 2 Division of Psychology & Mental Health, University of Manchester, Manchester, Greater Manchester, UK; 3 Manchester University NHS Foundation Trust, Manchester Academic Health Science Centre, Manchester, Greater Manchester, UK; 4 NIHR Greater Manchester Patient Safety Translational Research Centre, University of Manchester, Manchester, Greater Manchester, UK; 5 Division of Informatics, Imaging & Data Sciences, Centre for Health Informatics, University of Manchester, Manchester, Greater Manchester, UK; 6 Greater Manchester Mental Health NHS Foundation Trust, Prestwich, Manchester, UK

**Keywords:** Sedentary behaviour, physical activity, psychosis, mobile application, theoretical framework analysis

## Abstract

**Objective:**

To understand the acceptability of (a) reducing sedentary-behaviour in people with psychosis using ‘if-then’ plans and (b) the proposed app content.

**Design:**

Qualitative acceptability study.

**Method:**

Three structured focus-groups and an interview were conducted with eight participants who had experience of a psychotic episode. They discussed sedentary-behaviour, being more active, critical situations in which they may be tempted to be sedentary and solutions to these (the if-then plans), and a mock-up of the mobile application. The Theoretical Framework of Acceptability (TFA) was used to analyse qualitatively the transcripts.

**Results:**

All TFA constructs were coded in each of the transcripts. The idea of reducing sedentary-behaviour was acceptable to people with psychosis, participants knew the importance of being more active, however it is not always their main priority. Likewise, the proposed content of the app was found to be acceptable, with participants already using some of the proposed solutions.

**Conclusion:**

This was the first study to use the TFA framework to assess the acceptability of an app that uses critical situations and solutions (‘if-then plans’) to help reduce sedentary behaviour for people with psychosis. In this sample (male, English speaking mainly white people), participants understood the benefits of being more active. However, reducing sedentary-behaviour is not the main priority of this population and being sedentary has benefits when their mental-health is bad.

Individuals with a mental-health diagnosis, such as psychosis, die 10 to 20 years earlier compared to the general population due to conditions such as diabetes and cardiovascular disease (Ashdown-Franks et al., 2018). Many individuals with psychosis engage in more sedentary behaviours (11-hours; Stubbs et al., 2016) than the general population (5-hours; Townsend et al., 2015), which impacts negatively on health (Suetani et al., 2016). High levels of sedentariness interacts with mental-health and emotional outcomes (Hoare et al., 2016), increasing the risk of someone with psychosis experiencing emotional disorders (e.g. depression) and repeat psychotic episodes (Deighton & Addington, 2016).

The UK’s National Institute for Health and Care Excellence suggests that people with psychosis, especially those taking antipsychotic medication, need personalised programmes for reducing sedentary behaviour (National Institute for Health and Care Excellence [NICE], 2014). However, individuals may find it difficult to change their behaviour, due to barriers (e.g. stigma, medication side-effects and safety concerns; McDevitt et al., 2006; Ussher et al., 2007) that are not addressed directly in such programmes. Moreover, there are also issues in implementing behaviour change interventions for people with severe mental health issues in healthcare settings (Castaldelli-Maia et al., 2017, 2021). Further research is required to develop interventions that support people living with mental health conditions and can be used outside of healthcare settings. Volitional Help Sheets (VHS) provide a tool for people to identify personal barriers and develop solutions to overcoming them (Armitage, 2008). The VHS use ‘if-then’ plans (e.g. ‘*If* I’m inactive when I feel depressed *then* I will tell myself that being more active is part of my recovery’) to help individuals meet their goals (Gollwitzer, 1999; Sheeran et al., 2005), by creating personalised plans (Webb & Sheeran, 2007). ‘If-then’ plans work by identifying a salient situation (e.g. feeling depressed), and pairing it with a solution (e.g. reminding themselves of the benefits) so when that situation arises the appropriate solution for overcoming the barrier automatically links in the individual’s memory and the behaviour is more likely to be enacted (Arden & Armitage, 2012). The VHS is effective in those with mental-health issues (e.g. reducing self-harm and suicidal behaviour; Armitage et al., 2018), and is promising in managing emotional eating (Armitage, 2015). A meta-analysis found ‘if-then’ planning help people with mental-health conditions achieve goals (Toli et al., 2016); however, little research has been conducted in populations with psychosis. This research explores whether people with psychosis would find ‘if-then’ plans acceptable in encouraging them to be less sedentary, and is the first study to look at whether this activity would be acceptable in app format.

mHealth technology (e.g. mobile-phone apps), have reduced sedentary behaviour in individuals with mental-health problems; for example, text message prompts increased step-counts in those with severe mental-health problems (Chen et al., 2017), and a literature-review found that motivational/supportive text messages improved physical health in those with a psychotic disorder (Griffiths, 2020). Although, mHealth app research to reduce sedentary behaviour in those with psychosis is limited, apps are effective in increasing active behaviour in the general population (Conroy et al., 2014; Hollis et al., 2015; Vandelanotte et al., 2016). Furthermore, mHealth apps are already used by people with mental-health conditions including people with psychosis, for other purposes (e.g. symptom management, medication adherence; e.g. Careloop, 2022). Little research has looked at how these apps support behaviour change in people with psychosis. A smoking cessation app had little effectiveness as it was not tailored to people with psychosis (Ferron et al., 2017). Despite the prevalence of mHealth, there are barriers to developing apps that are acceptable for this population (Palmier-Claus et al., 2013). Research has shown symptoms (e.g. paranoia, disorganisation, cognitive impairment) may limit the acceptability and usage of apps for individuals with psychosis (Firth & Torous, 2015). Therefore, we need to understand what is acceptable with regards to apps from the population group themselves.

This study aims to explore (a) the acceptability of reducing sedentary behaviour and (b) the acceptability of an app for this purpose to people with psychosis. The results of this research will help to inform the development of apps that are tailored to this population.

## Methods

### Participants

Participants were recruited from organisations/forums for people who have experienced psychosis, the researchers’ contact list and citizen scientist groups. Inclusion criteria were self-reported psychotic episode (last 5-years), aged over 18, smartphone/tablet user with access to the internet, English speaking and in North West England. No one was excluded as all prospective participants met the eligibility criteria.

### Design and materials

The research team conducted three qualitative focus-groups and one semi-structured interview.

#### Topic-guide

The topic-guide (Supplementary materials) was iteratively changed depending on emerging topics. It covered four main topics: (a) understanding of sedentary behaviour (b) knowledge surrounding activity level and health, and thoughts on (c) pre-written and suggested ‘if-then’ planning items (Supplemental Materials) and, (d) a mobile app for making plans.

#### Mock-up of app

A mock-up of the app was shown to participants (Supplemental Materials) showing the proposed screens of the app, including planning items, enabling discussion around presentation.

#### Questionnaires

Measurements of physical activity (IPAQ, 2002), sedentary behaviour (Rosenberg et al., 2010) and technology use (Rosen et al., 2013) were taken.

### Procedure

Advertisements were placed on research/people with psychosis forums and organisations local to Manchester inviting interested individuals to contact the researcher for the participant information sheet. If interested, individuals were screened to ensure eligibility. Participants were allocated to a convenient focus-group at the University of Manchester. If only one person attended the focus-group, then an interview was conducted instead.

Two facilitators introduced the research and took informed consent. Participants completed questionnaires, discussed the topics with the aid of an example VHS and mock-ups of the app. Participants were debriefed, thanked for their time, and given monetary compensation (£25). Data were collected between July 2016 and April 2017.

### Data analysis

Each session was audio-recorded and transcribed. Both interviews and focus groups were analysed in the same way. Framework analysis was used to analyse, whereby data was coded deductively into the seven constructs of the Theoretical Framework of Acceptability (TFA) (Sekhon et al., 2017) with the extra construct of perceived appropriateness to identify individual’s personal opinions. The data analysis process involved three researchers (RB, KB, and TE) and a five stage process was used: familiarisation, identifying themes, indexing, charting/summarising, interpretation.

Familiarisation: this involved all three researchers reading through the transcripts.

Identifying themes: themes were already identified as we were using a pre-formulated framework (the acceptability framework - Sekhon et al. (2017)). During a discussion with the team about how the framework might relate to the data, each theme was further divided into sub-themes (i.e. for each of the acceptability framework items we looked generally at (a) physical activity and specifically at (b) the content and (c) the acceptability of the app). RB developed a coding manual that described each of the themes and subthemes. This was discussed and agreed up by the three researchers.

Indexing: RB coded all the data using the themes and subthemes in the coding manual. And noted any data that didn’t fit into those themes. These were discussed within the team and the coding manual was then updated to include the theme ‘Perceived appropriateness’. RB then coded the data using this additional code.

Charting/summarising: a matrix was formed by RB whereby illustrative quotes were selected and the data were summarised.

Interpretation: The research team met and discussed the data. The data was then fully interpreted by RB and drafts sent to the team for discussion until a final version was agreed upon.

For anonymity pseudo-identifiers were ascribed to participants (p1–p8).

The study received ethical approval from The University of Manchester Research Ethics Committee 2 (*Ref: ethics/16313*).

## Results

Focus-groups and interviews were carried out with eight participants. All participants were male, with an average age of 30.4-years. Their sedentary behaviour ranged from 3 to 38-hours per week (median 17.63). Their weekly median physical activity in hours was: walking 7.75, moderate 5.25 and vigorous 3. All participants had access to a smartphone/tablet and the internet. Six used their phone to text-message daily and two participants used their phone less frequently (Table S2 for participant characteristics). The data fit into the seven themes from the TFA (plus perceived appropriateness).

### Intervention coherence

Participants understood what sedentary behaviours were and listed sedentary behaviours that they performed. Sedentary behaviour was a positive in some circumstances (e.g. ‘chilling’, ‘relaxing’). Some distinguished between sedentary behaviour and physical activity and saw them as discrete things while others considered them to be on a continuum. Some regarded ‘being more active’ to reduce sedentary behaviour as moderate/vigorous exercise (e.g. gym, cycling) rather than incidental activity (e.g. regularly moving, housework, walking).

Participants understood the purpose of reducing sedentary behaviour (e.g. to improve mental-health). The intervention aligned with pre-existing knowledge of physical activity benefits *‘I’ve been generally told that if you stay active then your brain’s more active and it means that it tends to be healthier in general’* (p2).

Participants understood the purpose of the situations and solutions used in the app. Certain situations made sense to participants as there are already things that encourage them to be more active: *‘feeling bored, feeling bored is why I exercise’* (p6), ‘*I try and fight my negative thoughts, so that’s one reason to be physically active*’ (p5). Participants showed an understanding of how the app is *‘solving a problem’* (p6) and *‘the goal is to get people more active’* (p6). They understood the navigation of the app and its purpose of promoting a reduction in sedentary behaviour in people with psychosis.

### Affective attitudes

Participants discussed positive feelings about reducing their sedentary behaviour; and stated that physical activity improved their mental-health *‘I feel happy now cos I jogged here’* (p1), stress, anxiety and led to positive things overall *‘Exercise reduces stress. [. . .] if I feel worked up, or have something on your mind, jump on the bike or go to the gym or whatever’* (p6). Participants also discussed how too much inactivity could be negative *‘I sort of punish myself with the word (sedentary), because I feel as if I should be doing more’* (p5).

Participants felt positive about the app and thought that when they were in the right frame of mind (i.e. having the headspace to think about things other than their mental-health) it may be something that would encourage them to be more physical activity. *‘Yeah, it’s a positive thing what could help a lot of people if they’re not active’* (p3).

### Burden

Issues specifically related to psychosis and generic issues were identified around the burden of reducing sedentary behaviour. This was secondary to their mental-health therefore not their main priority; and when they were not well, an effort was required *‘you can’t really focus on physical exercise if your brain’s not in the right place’* (p2). Likewise, participants described how their medication *‘comes with side effects’* (p2) made activity burdensome *‘Some medication can make you not as active as you want to be’* (p3). Regarding app content, participants agreed with some situations on the app; for example, *‘it [being active] can be expensive, yeah’* (p1), how bad weather can *‘really put me off’* (p5) and when they are stressed as it is *‘totally overwhelming’* (p5). We did not specifically ask about the perceived burden of using the app but this was not bought up by the interviewees, suggesting that it would not require a lot of effort.

### Ethicality

Ethicality was coded when participants discussed their personal value systems. Participants in all focus-groups/interviews discussed the importance and benefits surrounding reducing sedentary behaviour and how they personally valued the benefits on mental-health *‘when I go the gym [. . .] I feel better, I feel my mind’s stronger’* (p1). Participants also talked about how they valued sedentary behaviour at certain times as *‘it could be relaxing [. . .] for a certain amount of time’* (p2) and it enabled them to *‘de-stress through those few hours that I can sit down and chill out, relax or whatever’* (p6).

Regarding the ethicality of the app, participants felt people would be more likely to use it if it was secure, *‘private, straight away, that’s one of the main things’* (p1). Individuals who experience psychosis may not want their diagnosis to be public knowledge, therefore expressed the need for the app have *‘a password on the app’* (p1) or *‘try and make it the same way as you unlock your phone’* (p2). The ethicality of the app content was discussed less, however both p1 and p6 made a strong point of how the solution, from the app, of *‘being a better role model’* related to their personal values and is a *‘big thing’* (p1) for them. They saw being more active as a positive behaviour for younger family members to see and would be a good solution to include *‘That specifically relates to my new nephew, if there’s something I can do to inspire him, great, obviously I want him to be physically active when he’s older’* (p6).

### Opportunity costs

Although there were few opportunity costs relating to being more active, p2 mentioned potential social disapproval in order to be active as there are individuals who may disapprove of them being more active especially if they were not active themselves.

This opportunity cost was also echoed in the situation included in the app content of being sedentary when spending time with family *‘If you want to spend time with your family, and all they do is sit down, you’re going to have to sit down. You can’t get them out on a treadmill can you?’* (p6). There was no indication among remaining interviewees that indicated there would be any opportunity costs regarding the app. However, as many had suggested that looking after mental-health was a priority over reducing sedentary behaviour there may be response costs of using the app in those circumstances.

### Perceived effectiveness

All participants perceived a positive relationship between being more active and their health *‘I feel more exercise can lead onto more positive things’* (p3) and doing low intensity activities such as yoga helped to *‘keep my mind calm’* (p8). Also discussing how being more active in general could be helpful when they might be feeling negative as *‘a way of [. . .] blocking out negative thoughts and to keep a productive day’* (p7). Whilst discussing the situations and solutions of the app, participants talked about which ones they personally related most to and would be effective, such as doing everyday tasks *‘Yeah I’ll do some washing or ironing, or something like that, or some DIY’*, or *‘walking the dog’* (p5). A solution that was discussed a lot was being active with a friend, for example having a gym buddy as it would help them as *‘friends are powerful’* (p1). However, friendships could have negative impacts on their activity levels if their friends were not active, *‘my friends are lazy pot-heads, so yeah, that’s not going to happen’* (p1) therefore, they recognised it may not be an effective solution for everyone. The idea of the situations and solutions on the app not being relevant to everyone was acknowledged *‘I think it’s a very individual thing, solutions, what works for one won’t work for others, and it’s a case of finding which one works for yourself’* (p5), therefore the situations and solutions from the app would not always be universally effective.

Participants perceived the app as a positive thing that could be encouraging as *‘it could help you, it’s hard to be motivated sometimes, so it’s good’* (p8). However, it was also felt as though the app would not *‘appeal to everybody’* (p7), and that there were certain situations, such as feeling down or negative, in which the app would not be effective (regardless of the content) *‘if they’re down and they’re in their room and they’re depressed and feeling rotten and they’ve woken up in a bad mood trust me nothing is getting them up’* (p1).

### Self-efficacy

Participants talked about their current levels of activity. Those that were active discussed how current factors in their life encouraged them to be more active *‘I’ve got a dog, and I’ve got a ten-month old nephew, I’ve not got the chance to [. . .] not do anything’* (p6). Although all participants wanted to be more active, there were factors that may conflict with their confidence in their ability to do so, for example their *‘social anxiety’* (p7) could be a barrier. Likewise, their mental-health can impose personal limits on activity levels thus reducing their self-efficacy regarding being more active *‘I can go so many days, and then I can collapse, well I can go flat, really tired, because basically, well you’ve done so much, now you’re going to suffer for it, the tiredness’* (p5).

Individuals showed confidence about engaging with the situations and solutions on the app. Listening to music was a popular and effective solution, *‘I listen to music and doing that motivates me to do more exercise’* (p3), showing confidence they could engage with this solution *‘[reading from sheet] “then I will put music on and go for a walk”. Now, that’s something that I used to do, and it sort of reminded me, that would be good to be a distraction for me as well at the time’* (p5). Participants also felt confident that reminding themselves physical activity is a part of their recovery would encourage them, as physical activity is a *‘massive part’* (p6) of recovery. Despite the participants’ confidence they could engage with the app’s situations and solutions, little confidence was expressed regarding app engagement when they are struggling with their mental-health, expressing how they would have *‘to be motivated’* (p1) themselves in order to engage.

### Perceived appropriateness

Overall participants opinions regarding reducing sedentary behaviour were positive, as it would help generate a sense of *‘purpose’* and give them something to *‘focus on’* (p2). Participants expressed how when they are more active it is beneficial in relation to their mental-health *‘personally, when I’m exercising, I feel back to how I was before the episode that I had [. . .] and I reflect on it quite a lot which develops quite a positive mind frame for myself’* (p6).

Mixed opinions were revealed regarding the situations and solutions of the app. P7 felt as though at some point during their life, they have or would find the individual situations and solution appropriate *‘I think each one of them describes a point I’ve been at in my life where I felt I can’t’*. A combination of different situations and solutions was suggested as more appropriate than just one by itself, *‘it’s like combinations of these things [. . .] it’s not just one of these by themselves. It’s these situations all kind of connect in different ways’* (p2). Opinions regarding the app varied throughout the transcripts, the participants liked the purpose and content. However, participants thought the design of the app *‘looked quite basic’* and *‘boring’* (p5) and proposed it would be more visually attractive if it was *‘more colourful, more vibrant, more appealing’* (p5). Some also felt the app needed to be more interactive in order to engage them, instead of just reading things off a screen

*“when I read something off a computer screen, even though it makes a valid point, it still has no meaning to me because another person has not said it. It’s got no actual emotional connection to it, [. . .] maybe if it could have [. . .] it read it out to you”* (p7). Tailoring the app to the individual was also something participants felt would be appropriate and more encouraging *“something tailored to the individual, I think that would allow people to feel a bit more able, a bit more comfortable to actually engage in physical activity”* (p6)

## Discussion

The Theoretical Framework of Acceptability was useful in determining the acceptability of reducing sedentary behaviour in people with psychosis, and the acceptability of using an app to do this. The data shows that the goal of the intervention to reduce sedentary behaviour in people with psychosis using an app incorporating VHS content delivered via an app was generally found to be acceptable. Participants recognised the health benefits of reducing sedentary behaviour, both in theory and practice, and identified that the intervention would be acceptable in relation to their personal values such as wanting to be a good role model and returning to normal life, expanding on current literature suggesting physical activity is beneficial for both physical and mental-health.

Whilst in the current literature sedentary behaviour is typically portrayed in a negative manner (Pearson et al., 2014), the analysis showed that those with mental-health conditions experience some positives of sedentary behaviour. Participants expressed that sedentary behaviour, at times, is a good thing helping them to manage their mental-health, for example, it gives them time to relax and de-stress.

The results also show that people with psychosis face specific challenges with regards to reducing sedentary behaviour due to factors relating to their mental-health; therefore, managing their mental-health comes first and decreasing their sedentary behaviour would not necessarily be a priority or appropriate for them at those times. At certain times, such as when they are feeling low, being more active may be prioritised differently, therefore the app may present conflicting goals in those situations. This indicates that the intervention could be suitable, but it would not be appropriate for use on those days when their mental-health was at its worst.

One strength of the app was that all participants viewed at least some of the proposed situations and solutions of the app as effective and welcomed the ability to exercise personal choice and personalising plans by selecting the ones that were most relevant to them. There were some proposed solutions to being more active – such as walking the dog or doing housework – that they already related to and already did. Individuals showed confidence that when they were managing their mental-health they would be able to engage in the situations and solutions on the app, and they viewed the app as an acceptable intervention with little effort to engage with. Despite the positive opinions regarding the app, participants did feel as though it would be more acceptable if the app included security features (e.g. password protection; PIN codes) to protect user confidentiality.

### Strengths and limitations

To our knowledge, this is the first study that has used the Theoretical Framework of Acceptability to explore the acceptability of reducing sedentary behaviour and the use of an app for this purpose for people with psychosis.^1^ The findings presented can help future researchers and developers to understand what would be required to develop apps that aim to reduce sedentary behaviour in this population. TFA was a useful framework for exploring acceptability, however there was some overlap between constructs such as Self-Efficacy and Ethicality, for example the solution of being a good role model increased individuals’ self-efficacy in engagement with the app, however this solution relates to the individual’s personal value system. Whilst appropriateness is mentioned in the framework’s definition of acceptability for this study it did not seem adequately covered in the seven constructs by Sekhon et al. (2017), therefore the researchers generated an additional code for ‘perceived appropriateness’ to understand individuals’ personal opinions of reducing sedentary behaviour using the app.

This was a small sample as the population is also small and difficult to recruit. Furthermore, participants were all male (despite efforts to recruit females) and the majority of the sample (seven out of eight) were White British, therefore, findings may not generalise to a broader demographic sample and women in particular. There may also have been a selection bias in that only those participants who were currently managing their psychosis well were able to take part – however, these participants did reflect back on times they were less in control of their condition. Focus-groups were the chosen method of data collection in order to capitalise on interactions between the participants; however, the interaction may have been limited due to the small number of participants in each group (this was due to availability of participants /drop outs and on one occasion an interview took place as there was only one person).

### Clinical implications and future research

This research has gained an insight into the acceptability of reducing sedentary behaviour in people with psychosis and whether this behaviour change app, and its proposed content is acceptable in the target population. Therefore, this research can help to aid in the development of such an app, and similar apps targeting changes to lifestyle behaviours in people with psychosis.

This research has only looked at the anticipated acceptability, measured before using the intervention. Sekhon et al. (2017) have suggested there might be differences between anticipated and experienced acceptability (measured either during or after using the intervention). Therefore, the next step would be to test such an app and VHS within this population to allow the collection of experienced acceptability data.

## Conclusion

An app that uses if-then plans to overcome barriers to reducing sedentary behaviour is seen to be acceptable by people with psychosis. However, the research has shown specific considerations that such an app would need to address to be suitable for people with psychosis. From the analysis, it is clear that whilst participants with psychosis know of the benefits of being less sedentary and more active when their mental-health is at its worse it is not their top priority, and sometimes being sedentary is seen as more beneficial. It is also important to be able to create a personal aspect of such a behaviour change app, so it is tailored to the individual and their situation. Such insight can help to design a behaviour change app to encourage this population to be more physically active. The next step in this research would be to use feasibility studies and a randomised controlled trial to assess whether ‘if-then’ planning would be acceptable in practice and encourage people with psychosis to reduce sedentary behaviour.

## Supplemental Material

sj-docx-1-isp-10.1177_00207640221102733 – Supplemental material for Acceptability of reducing sedentariness using a mobile-phone application based on ‘if then’ plans for people with psychosis: A focus-group study conducted in North West England, UK Click here for additional data file. Supplemental material, sj-docx-1-isp-10.1177_00207640221102733 for Acceptability of reducing sedentariness using a mobile-phone application based on ‘if then’ plans for people with psychosis: A focus-group study conducted in North West England, UK by Rachel Bailey, Y Kiera Bartlett, Lamiece Hassan, Christopher J Armitage, Charlotte Stockton-Powdrell, Matthew Machin, Shon Lewis and Tracy Epton in International Journal of Social Psychiatry

## Notes

1. Although, a survey version of the framework has been used to evaluate the acceptability of a training programme for teachers to deliver a physical activity intervention (Renko et al., 2020).
