# Supplementary material for: Acceptability of reducing sedentariness using a mobile-phone application based on ‘if then’ plans for people with psychosis: A focus-group study conducted in North West England, UK
Source: Int J Soc Psychiatry. 2022 Jun 7;68(5):1100–7. doi: 10.1177/00207640221102733 (PMC9310137; doi:10.1177/00207640221102733)
Supplement: sj-docx-1-isp-10.1177_00207640221102733 – Supplemental material for Acceptability of reducing sedentariness using a mobile-phone application based on ‘if then’ plans for people with psychosis: A focus-group study conducted in North West England, UK [file sj-docx-1-isp-10.1177_00207640221102733.docx]

**Supplementary Materials**

**Topic Guide**

Focus group guide

Complete consent forms

Questionnaire **10 mins (individually completed)**

Ensure all consent is correctly completed and begin recording

[Begin the group by introducing self, purpose of the group and history of the research]

e.g. Hi I’m ___ and I am a researcher here at the university, we also have xxx and xxxx in the room who are going to help me with this discussion. We’ve asked you here today because we are interested in your opinions about an app we are developing. We have been working with a few people with psychosis to develop these ideas but now we would like to see what a wider group think.

[Establish ground rules]

There are a few things I’d like to run through before we start. Firstly, there are no right or wrong answers, but rather differing points of view. We’d like you to feel free to share your point of view even it differs from what others have said. Sometimes somebody in the group will have a strong opinion about something and you may have the exact opposite opinion. We would like to hear whatever you have to say.

Keep in mind that we are just as interested in negative comments as positive comments, and sometimes the negative comments are the most helpful.

As we’re recording this discussion, please speak up and try not to speak over one another.

I’m going to ask you to say your name before you speak so I would say xxx then say what I was going to say. This is really helpful for us when we’re listening to the tape, but it can be difficult to get into the habit, I’ll try to remind you as we go through the group

We’ll only be using first-names here, and in our later reports there will not be any names attached to the comments. We do ask that you do not repeat anything you hear in this room.

My role here is to ask questions and listen, but I won’t be participating in the discussion. Feel free to talk with one another. There is a tendency in these discussions for some people to talk a lot and some people not to say much. But it is important for us to hear from each of you today because you have different experiences. So I may ask for an opinion from someone if they are not saying much, and move the discussion onto someone else if someone is sharing a lot, so we can make sure we hear from everyone.

We only have a limited time today and quite a lot to get through, so if someone is sharing something that is interesting, but not directly relevant to the questions we’re discussing I might suggest we park the idea, I’ll make a note, and if there is time at the end we can come back to it.

We’ll finish around xxx and at the end of the session, after you returned your completed questionnaire, we will give you £20 for your time, so make sure you don’t leave without it.

[Ice breaker, we usually ask people to go round the room introduce themselves by their first name and say what they like to do on the weekends, start with yourself and any staff members, then go round the group members]

FOCUS GROUP SCHEDULE

Activity 1: **10 mins (large group)**

To identify what is understood by sedentariness

Think about a typical week and the types of activities you tend to do.

1. What kind of activities do you do that involve you sitting down?

Prompt: Watching TV, video game playing, computer use, driving, reading, sewing, knitting, eating,

2. Which of these would you say also don’t take up a lot of energy?

Prompt: Watching TV, video game playing, computer use, driving, reading

3. What word / phrase would you use to describe these kind of activities?

Rephrase if needed : How would you describe activities that involve sitting down and don’t use a lot of energy, what word or phrase would you use?

4. Have you heard of the term sedentary behavior?

- - Give definition – any waking behavior involving low energy expenditure that is performed in a sitting or reclining position.

5. Can you think of another word for sedentary behavior?

- - E.g., inactive, getting up and doing something

Activity 2: **10 mins (large group)**

To identify knowledge about activity and mental and physical health

6. What, if any, do you think are the effects of spending a lot of time doing those kinds of low energy / sitting down activities?

- How, if at all, do you feel different when you are inactive and when you are active?

6b. What do you know about the risks of physical activity in people with psychosis?

6c What do your care workers think about you doing physical activity

Activity 3: **25 mins (large group)**

Thoughts on planning items

Loads of people have a goal of being less sedentary / more active but don’t always manage to do it because there are times when they don’t feel like it. Making plans on how to deal with times you don’t feel like it has been shown to help people be more active. We’re thinking of developing an app to help people make those plans

One way is to identify situations when you might be tempted to be inactive and make a plan of what you will do / tell yourself to do to make sure you are more active.

7. What do you think of these situations?

Prompts: Which, if any, are relevant to you?

8. What, if any, other situations would be relevant to you?

9. What do you think of these solutions?

Prompts: Which, if any, are relevant to you

10. We usually word them as IF – THEN. How, if at all, could we word them better?

We also use the tempted not to be physically active – do you think there is a better way of wording this so it is focused on not sitting down as much rather than going to the gym etc.

Activity 4: **25 mins**

Thoughts on the app

So we’ve spoken about making plans and the types of plans that might be relevant for you. Now we’d like to show you some early ideas we’ve had about an app to help people make these plans. As I said at the beginning we’re just as interested in negative comments and positive comments and we’re only very early on in the development, so whatever you have to say we would be happy to hear it. I’m going to run you through the whole app, then we’ll go back and speak about each screen.

[run through the app describing the process]

What, if any, do you think are the positive things about this app?

What if any do you think are the negative things about this app?

[Critical situations screens]

Here you can see the list of problems to choose from.

11. What do you think of this screen?

Prompt: look, language used, clarity

12. How, if at all do you think we could improve it?

[critical situation with confirmation]

Here you can see the solutions you’ve chosen before, and see the whole list if you want to

13. What do you think of this screen?

Prompt: look, language used, clarity

14. How, if at all do you think we could improve it?

When you choose a solution, you can see the whole statement and choose to add it, or change it.

15. What do you think of this notification?

Prompt: Clarity

16. How, if at all do you think we could improve it?

17. Thinking about the app overall, what, if anything could we do to improve this app?

Summary **10 mins** [ask participants and co-moderators if you have missed anything]

18. Does anyone have anything to add what we have discussed today?

If you are interested we can send you a report of what we’ve found from this and other focus groups. Let me know if you’d like this and I can email / post it out to you.

Thank participants.

**Volitional Help sheets – situations and solutions**

**SITUATIONS**

If I’m inactive when I’m under a lot of stress

If I’m inactive when I am depressed

If I’m inactive when I feel I don’t have the time

If I’m inactive because I am alone

If I’m inactive because I am spending time with family and friends who do not exercise

If I’m inactive because it is raining or snowing

If I don’t feel like being physically active because I don’t feel like

If I don’t feel like being physically active because it is cold outside

If I don’t feel like being physically active because I am overweight

If I don’t feel like being physically active because I feel tired

If I don’t feel like being physically active because of my negative thoughts

If I don’t feel like being physically active because I feel isolated

If I don’t feel like being physically active because I feel paranoid

If I don’t feel like being physically active because I have other commitments

If I don’t feel like being physically active because it is expensive

If I don’t feel like being physically active because my smoking makes it difficult

If I don’t feel like being physically active because I’m in a bad mood

If I don’t feel like being physically active because I’ve had an argument with family or friends

If I don’t want to exercise in a public place because I don’t understand the social rules

If I don’t want to exercise because am bored

If I don’t want to be active because of my side effects of my medication

**SOLUTIONS**

Then I will tell myself that if I try hard enough I can keep being physically active

Then I will put things around my home to remind me to be physically active

Then I will go out later in the day when I don’t feel tired

Then I will take someone with me when I exercise so I don’t feel intimidated

Then I will go to exercise at a quiet time

Then I will tell myself that physical activity is part of my recovery

Then I will do some gentle exercise like yoga or tai chi

Then I will remind myself that exercise reduces stress

Then I will exercise somewhere beautiful like the countryside

Then I will remind myself about the end result

Then I will do an exercise programme on DVD / YouTube / app

Then I will find cheap / free places or ways to exercise

Then I will put my music on and go for a walk

Then I’ll remind myself that even doing a little is better than nothing

Then I will think about information from articles and advertisements on how to make physical activity a regular part of my life

Then I will remember how warnings about the health hazards of inactivity move me emotionally

Then I will think how I would be a better role model for others if I were more physically active

Then I will tell myself that being more physically active would make me a healthier, happier person

Then I will make myself do some physical activity anyway because I know I will feel better afterwards

Then I will tell myself that I am being good to myself by taking care of my body in this way

Then I will seek out someone who encourages me to be physically active when I don’t feel up to it

Then I will tell myself that society is changing in ways that make it easier for people who want to be more physically active

Then I will do some star jumps

Then I will put my headphones in, my music on, and go for a walk

Then I will buy the right clothing to wear when it’s cold

Then I will do some jobs around the house to be more active

Then I will trick myself into exercise by doing something fun like swimming

Then I will tell myself I need to balance the things in my life and physical activity is part of that

Then I will take it one step at a time

Then I will tell myself it will make me feel more energetic

**Mock-up of the app**


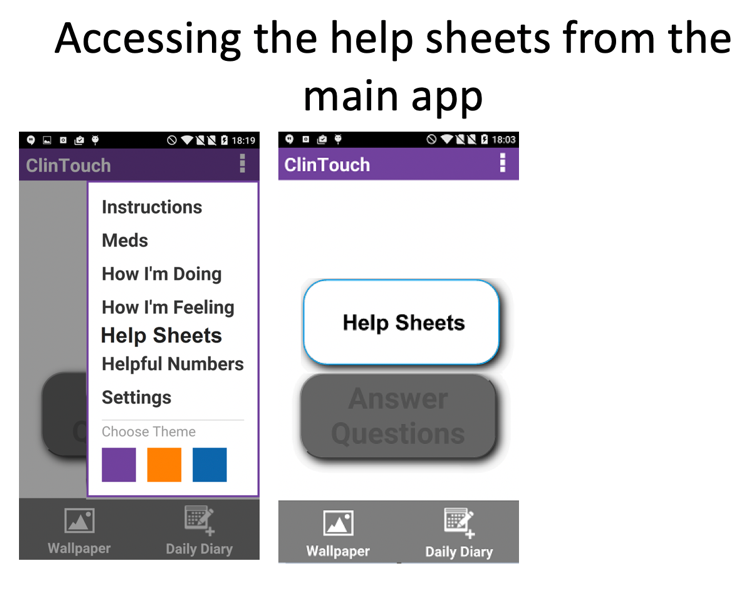

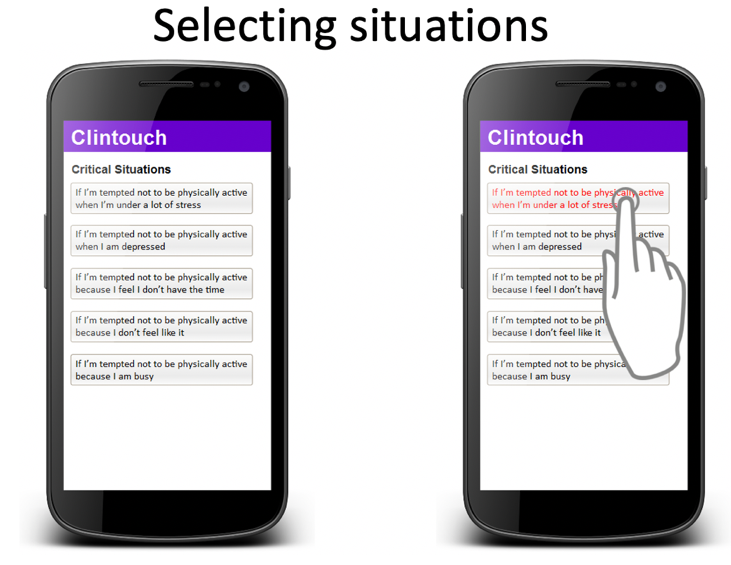

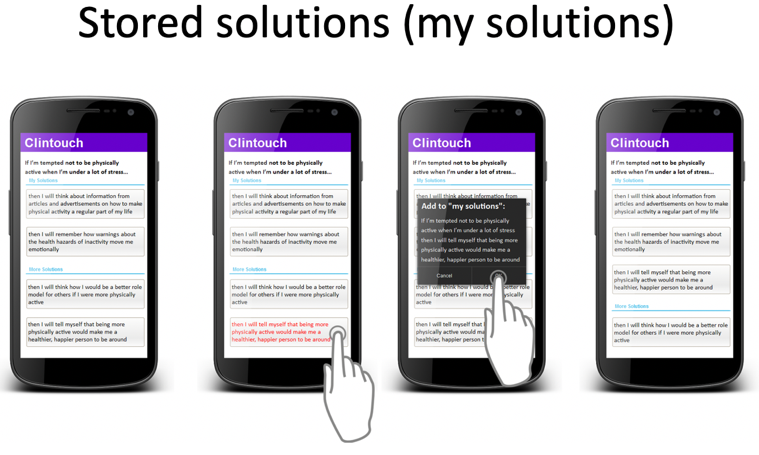

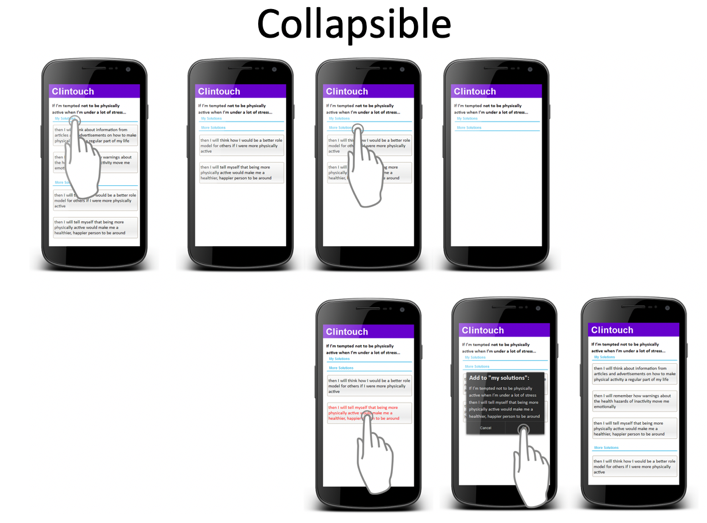

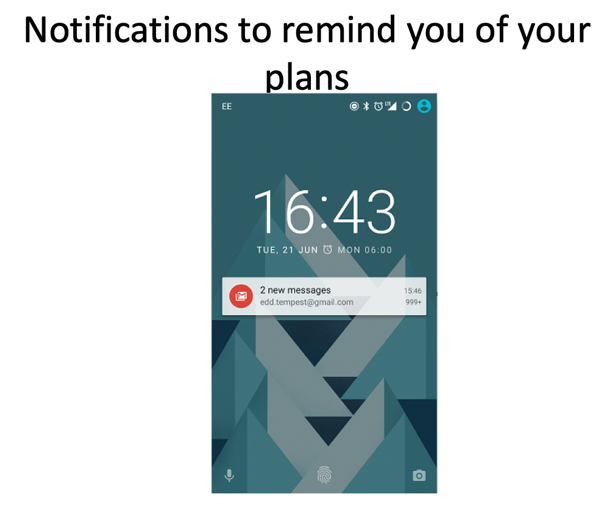


**Questionnaires**

Demographic questions were asked (i.e., gender, age, highest qualification, ethnicity, occupation, other medical conditions). The Sedentary Behaviour Questionnaire (Rosenberg et al., 2010), addressed how much time individuals on a typical weekday and weekend spend engaging in different sedentary behaviours (e.g. watching TV, sitting reading a book etc.). The International Physical Activity Questionnaire (2002) was used to measure physical activity of participants, asking how much they engaged in vigorous activity, walking and sitting in the last seven days. The Media and Technology Usage and Attitudes Scale looked at how much time individuals spend on their mobile-phones for certain activities (Rosen et al., 2013).

**Table S1. Coding Manual**

| **Theme** | **Code** | **Description** |
| --- | --- | --- |
| **Affective attitudes** | Regarding physical activity | How individuals feel about physical activity and their feelings about the benefits and advantages and disadvantages regarding their physical health and their mental health that result from being more active |
|  | Regarding the content of the app | How a participant feels about the different situations and solutions on the app, which situations they believe relate to them, and which solutions would be effective for them |
|  | Acceptability of the app | Individuals feelings on the app as a whole and the overall presentation of the app, whether it is motivating |
| **Burden** | Burden of physical activity | The amount of effort that is required to participate in physical activity. This includes the time, overcoming medication side effects, cost of joining a gym and having the correct equipment. |
|  | Burden of the app content | The amount of effort required by an individual to use the individual situations and solutions in the app |
|  | Burden related to the app | The burden perceived by individuals for using the app. |
| **Ethicality** | Ethicality regarding physical activity | How physical activity fits in with their personal value systems and why being more physically active may be a positive for them – if they don’t value PA then do they value sedentary behaviour |
|  | Ethicality regarding the app content | How situations and solutions in the app relate to individuals value systems for example the idea of being a better role model for others if they were active |
|  | Ethicality of the app | How the app itself fits in with individuals personal value systems – important issue brought up is the value of privacy of information stored on the app |
| **Intervention coherence** | Intervention coherence regarding physical activity | The extent to which participants understand being physically active and the effect it may have on their mental health at the time |
|  | Intervention coherence regarding the app content | The extent to which individuals understand the situations and solutions and how they work |
|  | Intervention coherence regarding the app | How well individuals understand the overall aim and navigation of the app |
| **Opportunity costs** | Opportunity cost of physical activity | The extent to which individuals feel profits or values need to be given up in order to engage in physical activity; e.g., they need to put things such as their mental health before physical activity |
|  | Opportunity cost of the app content | The extent to which individual feel they need to give up profits or values to engage in certain solutions in the app. For example, family or friends may not be physically active, therefore in order to be more active they may not spend as much time with other individuals |
|  | Opportunity cost of the app | The extent to which individuals feel they need to give up profits or values to engage with the app |
| Perceived effectiveness | Perceived effectiveness of physical activity | The extent to which individuals perceive physical activity will improve their health – and other benefits e.g. break apart from unhelpful friendship groups |
|  | Perceived effectiveness of the app content | The extent to which individuals personally feel the situations and solutions in the app will increase their physical activity |
|  | Perceived effectiveness of the app | The extent to which individuals believe using the app will increase their physical activity |
| Self-efficacy | Self-efficacy regarding physical activity | Individuals confidence that they can engage in more physical activity |
|  | Self-efficacy regarding the app content | Individuals confidence that they can use the situations and solutions in the app. For example, things like listening to music may be something they already enjoy therefore would help them engage in the behaviour |
|  | Self-efficacy regarding the app | Individuals self-confidence that they can engage in the app. Whether they would be able to engage in the app or if they are feeling low an app wouldn’t motive them. |
| Perceived appropriateness | Regarding physical activity | Individuals opinions regarding physical exercise, whether they see it as something that will be good or bad in relation to their mental health |
|  | Regarding the app content | Individuals opinions of the situations and solutions, whether they think they will be appropriate for them |
|  | Regarding the app | Individuals opinions on the app overall, is the presentation and overall look good or bad |

**Table S2. Participant Characteristics**

| Demographics | | | | | | | IPAQ | | | | | | | SBQ | | | |  | |  | |  | |
| --- | --- | --- | --- | --- | --- | --- | --- | --- | --- | --- | --- | --- | --- | --- | --- | --- | --- | --- | --- | --- | --- | --- | --- |
| ID | Age | Gender | Qualifications | Ethnicity | Occupation | Other medical conditions | Vigorous  (day x hrs) | Moderate  (day x hrs) | Walk  (day x hrs) | | Sedentary (hrs) | | Weekday (hrs) | | Weekend (hrs) | | Devices | | texts | | Internet | |  |
| 1 | 22 | M | None | White British | Unemployed |  | 1 day  3 hrs | 3  2hrs & 30 mins | | 7 days  6 hrs | | 7 | | 16.5 | | 12 | | Mobile (not smart)  Tablet | | >10 send  > 10 receive | | Yes | |
| 2 | 21 | M | degree | White British | Technical / IT |  | 2 days 1.5 hrs | 1 day  1 hr | | 4 days  2 hrs & 45 mins | | 5 | | 5.5 | | 9.75 | | Smartphone  Tablet | | 2-9 send  2-9 receive | | Yes | |
| 3 | 31 | M | NVQ / BTEC | White British | Unemployed |  | 3 days  1 hr | 3 days 1.5 hrs | | 3 days  30 mins | | 4.5 | | 12.5 | | 14.75 | | Smartphone | | 2-4 send  2-4 receive | | Yes | |
| 4 | 34 | M | Degree | Asian / Asian British | Technical / IT  Self employed |  | 3 days  2 hrs | 3 days  2 hrs | | 5 days  20 mins | | 20 | | 24 | | 14 | | Tablet | | <=1 send  <=1 receive | | Yes | |
| 5 | 48 | M | Degree | White British | Production / Manufacturing | Depression / high cholesterol | 1 day  2 hrs | 0 days | | 2 days  2 hrs | | 8 | | 7.25 | | 7.75 | | Smartphone | | >10 send  > 10 receive | | Yes | |
| 6 | 29 | M | Degree | White British | Technical / IT |  | 3 days  3 hrs | 1 day  1 hrs & 30 mins | | 7 days  4 hrs & 30 mins | | 6 | | 5.5 | | 6.5 | | Tablet | | 2-9 send  2-9 receive | | Yes | |
| 7 |  | M | A level | White British | Unemployed |  | 7 days  3hrs & 30 mins | 7 days  4 hrs & 40 mins | | 7 days  2 hrs & 20 mins | | 3.5 | | 9 | | 11 | | Smartphone | | >10 send  > 10 receive | | Yes | |
| 8 | 28 | M | International Baccalaureate | White British | Other employment |  | 0 days | 5 days  2 hrs & 30 mins | | 5 days  2 hrs | | 10 | | 2 | | 1 | | Tablet | | 2-9 send  2-9 receive | | Yes | |

M = male; IPAQ = International Physical Activity Questionnaire; hrs = hours; SBQ = sedentary behaviour questionnaire
